# Supplementary figures and images for: Role of Tobramycin in the Induction and Maintenance of Viable but Non-Culturable Pseudomonas aeruginosa in an In Vitro Biofilm Model
Source: Antibiotics (Basel). 2020 Jul 10;9(7):399. doi: 10.3390/antibiotics9070399 (PMC7400124; doi:10.3390/antibiotics9070399)

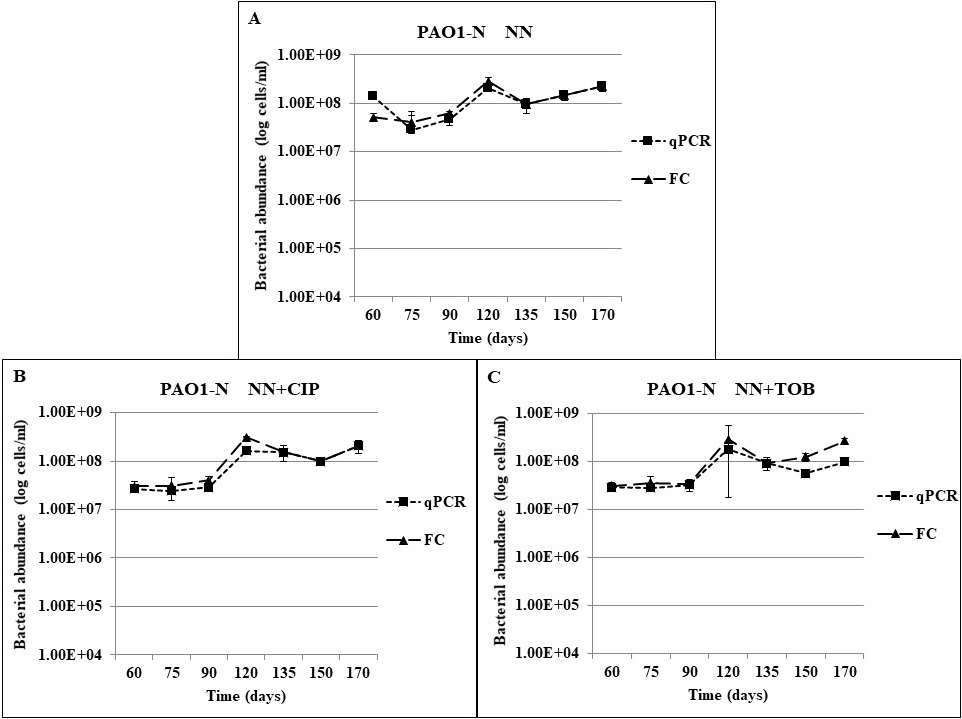

Supplement: Supplementary file 1 [file antibiotics-09-00399-s001.zip › Figure S1 300 dpi.jpg]
